# Biocontrol-relevant diversity of wheat-associated *Pseudomonas*: prevalence of *P. sivasensis* and identification of the novel species *P. arvensis* sp. nov.

**DOI:** 10.7717/peerj.20177

**Published:** 2025-11-05

**Authors:** Mathieu Delitte, Benjamin Dubois, Louis Morandini, Alain Bultreys, Jacques Mahillon, Frederic Debode, Claude Bragard

**Affiliations:** 1 Bioengineering Unit, Life Sciences Department, Walloon Agricultural Research Center, Gembloux, Belgium; 2 Earth and Life Institute—Applied Microbiology, Plant Health, UCLouvain, Louvain-la-Neuve, Belgium; 3 Earth and Life Institute—Applied Microbiology, Food and Environmental Microbiology, UCLouvain, Louvain-la-Neuve, Belgium; 4 Crops and Forest Health Unit, Life Sciences Department, Walloon Agricultural Research Center, Gembloux, Belgium

**Keywords:** *Pseudomonas sivasensis*, *Pseudomonas arvensis* sp. nov., Wheat, Phytobiome, Biocontrol, *Zymoseptoria tritici*, Whole genome sequencing

## Abstract

The role of microbial communities in plant health and productivity has become increasingly evident. In this study, we investigated the diversity and functional potential of *Pseudomonas* spp. associated with Belgian cereal crops, focusing on wheat and spelt. From 444 *Pseudomonas* isolates, we identified 11 species, with several strains exhibiting strong antagonistic activity against major wheat pathogens. Notably, we report the high prevalence and efficient colonization abilities of *Pseudomonas sivasensis*, including its capacity to translocate from seed to leaf and suppress *Zymoseptoria tritici* infection* in planta*. Furthermore, we describe *Pseudomonas arvensis* sp. nov., a newly characterized species within the wheat microbiome, with DR1PS3 designated as the type strain. Our results advance the understanding of beneficial plant-microbe interactions in cereals and highlight the promise of native *Pseudomonas* species as biocontrol agents for sustainable disease management.

## Introduction

Wheat is a major component of global agriculture, playing a vital role in ensuring food security and nutrition worldwide. Its widespread use and ability to thrive in various environments have established it as a dietary staple globally, contributing significantly to the economic and cultural landscape of societies. Despite its importance, wheat cultivation faces major threats from pathogens like *Zymoseptoria tritici*, the causative agent of Septoria Tritici Blotch (STB), which poses challenges due to its evolving resistance to fungicides (Fones & Gurr, 2015).

The microbial community within the wheat phytobiome plays a critical role in nutrient cycling, growth enhancement, and particularly in disease resistance. This complex system, consisting of the rhizosphere, phyllosphere, and endosphere, interacts dynamically with the plant, significantly influencing its health and productivity (Bulgarelli et al., 2012; Lundberg et al., 2012; Turner, James & Poole, 2013; Zhang et al., 2021; Lengrand et al., 2024). Understanding this intricate microbiome is key to developing sustainable disease management strategies. Recent studies have described these wheat leaves associated communities including fungi, bacteria and viruses. Common fungal taxa include *Zymoseptoria tritici, Blumeria graminis, Fusarium* spp., *Alternaria* spp., and *Cladosporium* spp. (Bensch et al., 2012; McDonald & Stukenbrock, 2016; Parry, Jenkinson & McLeod, 1995; Rotem, 1994). Among bacteria, *Pseudomonas* spp., *Pantoea* spp., *Bacillus* spp., *Methylobacterium* spp., and *Sphingomonas* spp. are frequently found (Kavamura et al., 2021; Li et al., 2023). Viruses and bacteriophages reported in association with wheat leaves are almost exclusively pathogenic, as they are typically investigated due to their detrimental effects. These include Hamiltonella virus (Forero-Junco et al., 2022), wheat mosaic virus (WMoV) (Tatineni & Hein, 2021), wheat yellow mosaic virus (WYMV) (Wang et al., 2024), wheat streak mosaic virus (WSMV) (Singh et al., 2018), and wheat dwarf virus (WDV) (Pfrieme et al., 2023).

Among the microbial constituents, *Pseudomonads* have been highlighted as keystone species in the wheat microbiome, crucial for their biocontrol properties and overall plant health (Sivakumar et al., 2020; Kumar et al., 2021).

This study seeks to investigate the biocontrol potential of *Pseudomonas* species associated to wheat. We aimed to understand how these bacteria colonize the plant, their prevalence, and their antagonistic effects against major wheat pathogens, contributing to sustainable agricultural practices through the use of native biocontrol agents. Our investigation led to the discovery of a new *Pseudomonas* species, *Pseudomonas arvensis* sp. nov., enhancing our taxonomic and ecological knowledge of the genus. This discovery underscores the importance of advanced taxonomic tools in identifying and characterizing microbial species within the cereal microbiome. This study also marks the first reported association of *P. sivasensis* with cereal crops, significantly broadening our understanding of its ecological range and biocontrol potential.

## Methods

### Bacterial strains selection

Four cereal fields in Belgium (Table 1) were sampled in 2019 at four periods (ends of March, April, May, and June) to collect *Pseudomonas* isolates from wheat heads, leaves, roots, and loose soil. These were obtained by single colony isolation on CFC agar (cephaloridine, fucidin, and cetrimid), a medium designed for *Pseudomonas* growth. Isolates were preserved in lysogeny broth (LB) medium supplemented with 20% glycerol.

**Table 1:** Characteristics of the sampled fields.

**Field**	**Field**	**GPS**	**Host**	**Agricultural**	**Tillage**
**Letter**	**Location**	**Coordinates**		**Practices**	
A	Jemeppe-sur-Sambre	50°29′47.0″N 4°37′32.6″E	Winter wheat	Organic	Yes
B	Jodoigne	50°43′13.4″N 4°51′22.4″E	Winter wheat	Conventional	Yes
C	Walhain	50°39′22.2″N 4°38′52.3″E	Spelt	Organic	No
D	Mont-Saint-Guibert	50°39′08.2″N 4°38′20.4″E	Winter wheat	Conventional	No

To select strains with biocontrol potential, a rapid prescreening was performed by qualitative observation of direct *in vitro* antagonism against *Fusarium graminearum*, adapting antibiosis tests described for toxins (Bultreys & Gheysen, 1999), but using LB agar. Briefly, a mycelial plug was placed at the center of the plate and 20 µl drops of 24 h bacterial cultures were placed at 2.5 cm of the plug. Plates were then incubated at 22 °C for 7 days. In a second step, their ability to antagonize four wheat fungal pathogens was assessed through *in vitro* confrontation with a quantitative approach, focusing on the mycelial growth reduction in *F. graminearum* (causing head blight), *Gaeumannomyces graminis* var. *tritici* (causing take-all), *Oculimacula yallundae* (causing eyespot), and *Z. tritici* (causing STB). For the first three fungi, an agar piece from a one-week-old fungus culture grown on potato dextrose agar was placed at the center of a LBA plate. Subsequently, three 10 µl drops of a 24-h LB liquid culture (30 °C, 180 rpm) of the bacteria were introduced at 2.5 cm from the center in three opposite directions. The plates were then incubated at 22 °C for 7 days (*Fusarium graminearum*) or 12 days (*Gaeumannomyces graminis* var. *tritici* and *O. yallundae*) and the inhibition zone radius was measured at the end of the incubation period.

Considering the slow mycelial growth of *Z. tritici*, an alternative top-agar method was used for evaluating the strain antagonistic ability against this pathogen (Kauffman & Polz, 2018). Briefly, conidia from a 10-day old *Z. tritici* LB liquid culture (22 °C, 150 rpm) were harvested, adjusted to 10^6^ spores/mL. One milliliter of the spore suspension was mixed with freshly autoclaved LBA at 45 °C and immediately spread over a previously constituted thin PDA plate. When solidified, a 10-µl drop of a bacterial suspension, prepared as described earlier, was placed at the center of the plate. The inhibition zone radius was measured 10 days later. All antagonism tests were conducted in three biological repetitions and in two temporal repetitions. Statistical analyses were performed in RStudio (*v.* 4.3.3) using t.test of the stats package.

### Bacterial strain initial identification

Following a 24-h incubation in LB medium (yeast extract, 5 g/L, NaCl, 10 g/L, bacto tryptone, 5 g/L) at 28 °C and 150 rpm, the bacterial suspensions underwent centrifugation, and the resulting pellets were used for DNA extraction using the Invitrogen PureLink Genomic DNA Kit (Fisher Scientific, USA) in accordance with the manufacturer’s recommendations. Molecular identification involved a Multi-Locus Sequence Analysis (MLSA) targeting 10 genes: 16S rRNA, *gltA, rpoB, glnS, gapA, gyrB, ileS, nuoD, recA* and *rpoD* genes. PCR amplification conditions were as follows: initial denaturation at 94 °C for 5 min, 30 cycles of denaturation at 94 °C for 30 s, annealing for 30 s, elongation at 72 °C for 60 s, and a final extension step at 72 °C for 5 min. The characteristics of primers and their annealing temperatures are detailed in Table 2. Amplicon length was checked on agarose gel. Sequencing was conducted by Eurogentec (Belgium) using Sanger sequencing. Subsequently, sequences were concatenated and queried against the RefSeq Representative Genomes database at the National Center for Biotechnology Information (NCBI).

**Table 2:** Characteristics of primers and annealing temperature.

**Target gene**	**Primer name**	**Sequence 5′–3′**	**Amplicon** ** size (bp)**	**Annealing** ** temperature (°C)**	**Reference**
16S rRNA	27F	AGAGTTTGATCCTGGCTCAG	1,400	51	Richert, Brambilla & Stackebrandt (2005)
	1492R	GGTTACCTTGTTACGACTT			
Citrate synthase (*gltA*)	gltA_F	AGTTGATCATCGAGGGCGCWGCC	529	60	Nikolić et al. (2018)
	gltA_R	TGATCGGTTTGATCTCGCACGG			
DNA-directed RNA polymerase subunit beta (*rpoB*)	LAPS5	TGGCCGAGAACCAGTTCCGCGT	1,230	50	Nikolić et al. (2018)
	LAPS27	CGGCTTCGTCCAGCTTGTTCAG			
Glutaminyl-tRNA synthetase (*glnS*)	glnS_F	ACCAACCCGGCCAAGAAGACCAGG	710	60	Andreani et al. (2014)
	glnS_R	TGCTTGAGCTTGCGCTTG			
Glyceraldehyde-3-phosphate dehydrogenase A (*gapA*)	GapA_F	CGCCATYCGCAACCCG	476	60	Andreani et al. (2014)
	GapA_R	CCCAYTCGTTGTCGTACCA			
Gyrase subunit B (*gyrB*)	GyrB-2F	ACCGTCGAGTTCGACTACGA	1,461	57	Andreani et al. (2014)
	GyrB-4R	CCTCGGTGTTGCCSARCTT			
Isoleucyl-tRNA synthetase (*ileS*)	ileS_F	TTCCCAATGAARGCCGGCCTGCC	633	60	Andreani et al. (2014)
	ileS_R	GGGGTGGTGGTCCAGATCACG			
NADH dehydrogenase subunit D (*nuoD*)	nuoD_F	GAAGTCCTGACCTTCCTGC	771	60	Ait Tayeb et al. (2005)
	nuoD_R	GAAGAACTCGGCCATCATG			
Recombinase A (*recA*)	recA_F	TGGCTGCGGCCCTGGGTCAGATC	537	60	Mulet et al. (2009)
	recA_R	ACCAGGCAGTTGGCGTTCTTGAT			
RNA polymerase sigma factor (*rpoD*)	PsEG30F	ATYGAAATCGCCAARCG	743	55	Mulet, Lalucat & García-Valdés (2010)
	PsEG790R	CGGTTGATKTCCTTGA			

### *In vitro* characterization

To evaluate the cellulolytic activity of the bacterial strains, the method described by Thomloudi et al. (2021) was followed. Briefly, 10 µL of bacterial liquid culture was placed in the center of a Petri dish containing the carboxymethyl cellulose (CMC) medium. After 7 days of incubation at 37 °C, plates were flooded with two mL of Lugol’s solution. Cellulolytic activity was measured by the size of the translucent halo around the bacterial colonies. Proteolytic activity was assessed using the method outlined by Durham, Stewart & Stellwag (1987). Briefly, bacterial suspensions were inoculated on plates containing the skimmed milk medium. After overnight incubation at 30 °C, activity was measured by the size of the translucent halo surrounding the bacterial colonies. Siderophore production was evaluated as explained by Bultreys & Gheysen (2000), but using King B agar. The orange halo around the bacteria after 24 h of incubation at 30 °C indicated siderophore production. Swimming and swarming motility assays were conducted following Thomloudi et al. (2021). Briefly, 10 µL of overnight bacterial culture was inoculated on NA medium amended with 0.3% and 0.5% agar, respectively. Petri dishes were incubated at 28 °C for one day. Biofilm formation was evaluated using the 96-well PVC plate assay as described by O’Toole (2010) and Thomloudi et al. (2021). Briefly, diluted (1:100) overnight bacterial cultures (100 µL) were inoculated in each well and incubated at 28 °C for 2 days. Wells were washed three times with distilled water before adding 200 µL of crystal violet. After incubating at room temperature for 30 min, the biofilm was solubilized with a solution of 20% ethanol and 80% acetone and measured by the intensity of the purple color. All tests were conducted in three biological repetitions and in three temporal repetitions.

### DNA extraction, genome sequencing, assembly, annotation and mining

DNA was extracted from pelleted 24 h bacterial cultures using the NucleoSpin Soil kit (Macherey-Nagel, London, UK), with SL1 and SX as lysis solutions. After purification with AMPure XP beads, the quality of the DNA was checked on a 0.8% agarose gel and with a Nanodrop spectrometer (Thermo Fisher Scientific, Waltham, MA, USA). The DNA concentration was assessed using a Qubit 4 fluorometer (Thermo Fisher Scientific). Sequencing libraries were constructed with the Ligation Sequencing Kit V14 (SQK-LSK114; Oxford Nanopore Technologies, Oxford, UK) following the manufacturer’s recommendations. Each library was loaded on an R10.4.1 Flongle flowcell, and a MinION device from ONT (Oxford Nanopore Technologies) was used to carry out the sequencing for 24 h. For the CF10PS3 strain, an additional WGS assay was conducted using Illumina technology to allow a (near-)perfect hybrid genome assembly using both ONT and Illumina sequencing data. For this trial, the extracted DNA was sent to Eurofins Genomics (Constance, Germany) to be sequenced on an Illumina NovaSeq device using the NovaSeq 6000 S4 PE150 XP sequencing mode.

The raw ONT sequencing data (POD5) was basecalled using Dorado (v0.3.4) with the basecalling model dna_r10.4.1_e8.2_400bps_sup@v4.2.0. Adapters were removed using Guppy (v6.5.7) and genomes were then assembled using Trycycler (v0.5.4) (Wick et al., 2021). For the CF10PS3 strain, an ONT + Illumina hybrid assembly was performed following the approach described in Wick, Judd & Holt (2023). Briefly, Trycycler was used to build a long-read assembly, which was then polished using Medaka (v1.8.0) (Oxford Nanopore Technologies, 2023). This assembly without structural errors was then polished with Illumina data using Polypolish (v0.5.0) (Wick & Holt, 2022) and Polca (Zimin & Salzberg, 2020) to remove remaining small-scale errors.

The assembled genomes were then annotated using the Prokaryotic Genome Annotation Pipeline (PGAP) (Li et al., 2021). Functional pathways were retrieved using the Kyoto Encyclopedia of Genes and Genomes (KEGG) (Ogata et al., 1999) and the KEGG Automatic Annotation Server (Moriya et al., 2007). To evaluate the strain potential for secondary metabolite production, AntiSMASH (Blin et al., 2023) and the virulence factor database (VFDB) with VFanalyser (Liu et al., 2022) were used. VFanalyzer constructs orthologous groups within the query genome and pre-analyzed reference genomes from VFDB to avoid potential false positives due to paralogs, then conducts iterative and exhaustive sequence similarity searches among the hierarchical pre-build datasets of VFDB.

To confirm MLSA identification of the strains with a whole genome-based taxonomic analysis, genome sequence data were uploaded to the Type (Strain) Genome Server (TYGS), a free bioinformatics platform available at https://tygs.dsmz.de, accessed on 27 March 2024 (Meier-Kolthoff et al., 2022). Information on nomenclature, synonymy, and associated taxonomic literature was provided by TYGS’s sister database, the List of Prokaryotic names with Standing in Nomenclature (LPSN, available at https://lpsn.dsmz.de) (Meier-Kolthoff & Göker, 2019). TYGS analysis involved using the MASH algorithm (Ondov et al., 2016) and 16S rDNA gene sequences extracted using RNAmmer (Lagesen et al., 2007) to determine the closest type strain genomes. Digital DNA-DNA Hybridization (dDDH) values were computed using the GGDC 4.0 method (Meier-Kolthoff et al., 2013). A balanced minimum evolution tree with branch support was inferred using FASTME 2.1.6.1 (Lefort, Desper & Gascuel, 2015) and visualized with PhyD3 (Kreft et al., 2017). Type-based species and subspecies clustering was performed using a 70% dDDH threshold (Meier-Kolthoff & Göker, 2019). In parallel, average nucleotide identity (ANI) was calculated with orthoANIu method, considered as the most accurate method (Yoon et al., 2017), with a threshold for species delineation set at 96% (Madhaiyan et al., 2017).

### Plant colonization

Transformation to express Green Fluorescent Protein (GFP) was performed following the recommendations of Choi & Schweizer (2006). Briefly, plasmids were obtained from an overnight liquid culture (37 °C, 220 rpm) of *Escherichia coli* strain DH5*α* containing either *Tn7::mNeonGreen* plasmid or helper plasmid *pTNS3*. Culture was conducted in LB supplemented with 10 µg/mL gentamycin and 0,2 mM diaminopimelate (DAP). Plasmids were extracted with QIAprep Spin Miniprep kit (Qiagen) following manufacturer recommendations. To prepare competent cells, strains in the exponential growing stage were washed three times in a sucrose solution (102.7 g/l) and transformed by electroporation. Briefly, 100 µl of competent cells were mixed with 500 ng of *Tn7::mNeonGreen* and 500 ng of helper plasmid *pTNS3* and electroporated at 2.5 kV. Transformed cells were spread on LBA plates containing gentamycin antibiotic.

Bacteria expressing GFP were then cultivated in 50 mL Falcon containing 20 mL of LB for 24 h (30 °C, 180 rpm) before being washed twice and resuspended in sterile MgCl_2_ + 1% Tween20 at a 10^8^ CFU/mL concentration. Twenty seeds from the Chevignon wheat cultivar were soaked in this suspension for 1 h before sowing in an autoclaved 70/30 mix of loam/perlite. Plants were maintained in a growth chamber with a day/night cycle of 8 h light and a temperature of 20 °C. Hydathode morning dew and total leaves were harvested 9, 15 and 30 days after sowing. Drops exiting hydathodes were immediately spread on LBA plates supplemented with gentamycin and CFC plates.

The following detached leaves protocol was applied to follow the epiphytic spatial distribution of the strain expressing GFP. Wheat seeds of the Chevignon cultivar, showing intermediate resistance to *Z. tritici*, were grown in an autoclaved 70/30 mix of loam and perlite in plastic trays in a glass-house with 16 h of light and a constant temperature of 22 °C until the second leaf was fully expanded. Water agar (15 g/L) containing 100 mg/L benzimidazole (Sigma), used to retard senescence, was dispensed into sterile clear polystyrene boxes (10 × 10 cm) (Mishra & Misra, 1973). Two rectangular sections (2.5 × 10 cm) were cut from the center of the agar. The seedling second leaf sections were laid, top surface uppermost, across the gap so that the cut ends remained on the agar. The gap below the leaves helped to prevent water soaking and contamination by other microorganisms. In this gap, a sterile paper piece soaked with one mL sterile water was introduced to maintain high humidity in the box. Eight leaf sections were fitted into each box. Strips of agar were then laid over the cut edges of the leaf sections so that they were not exposed, thereby delaying senescence. Bacterial suspension was constituted with a 24 h LB culture washed and resuspended in MgCl_2_ + Tween20 at a 10^8^ CFU/mL concentration. In each box, 2 ml of this suspension was applied. Leaf surfaces were observed under a laser confocal microscope after inoculation and every 5 days for up to 40 days.

### Plant protection

To evaluate the ability of the strains to protect the plant by reducing STB infection and its pycnidia production, a detached leaves protocol was designed. Boxes with detached leaves and their inoculation with bacterial suspensions were performed as explained in the last paragraph. All boxes containing detached leaves were constituted on the first day, whereas bacterial and mock treatments (treated with the MgCl_2_ + Tween20 buffer only) took place that same day, the day after, and three days later. After bacterial inoculation on the last day, boxes were left for 30 min under a laminar flow to allow leaves to dry before pathogen application.

Fungal inoculum of *Z. tritici* was produced from an active culture grown on LBA. A small piece of actively growing mycelium cut from the edge of a fungal colony derived from a single spore was placed in 10 mL of LB in a 50 mL Falcon tube. After 10 days of incubation at 22 °C under shaking, spores were harvested by centrifugation and washed twice in sterile MgCl_2_ + 1% Tween 20. The suspension concentration was then adjusted to 10^6^ spores/mL. In each box where bacteria had been applied two or three days before, or 30 min earlier, two mL of this suspension was applied, and boxes were closed and incubated at 20 °C with a light/dark period of 8/16 h. Symptomatic area and number of pycnidia were scored 28 days after inoculation. All assessments were carried out using a dissecting microscope at 40X magnification. Statistical analysis was performed in RStudio (v. 4.3.3) using t test of the *stats* v4.3.3 package.

## Results

### Isolates characterization

From the 444 *Pseudomonas* isolates obtained at the end of the sampling, an initial qualitative screening identified 114 isolates that exhibited direct antagonism against *F. graminearum*. These 114 isolates were subsequently tested in quantitative assays, measuring their ability to inhibit mycelial growth of four wheat pathogens. Among them, 33 isolates displayed strong antagonistic activity against at least three of the four pathogens (Table 3). They were all active against the two soilborne pathogens *G. graminis* var. *tritici* and *O. yallundae*, and showed various abilities to antagonize *F. graminearum* and *Z. tritici*. For isolates obtained from heads, 11/12 were able to control *F. graminearum*. Similarly, 5/6 foliar isolates are able to antagonize *Z. tritici*.

**Table 3:** *In vitro* antagonist activities of the *Pseudomonas* isolates and their bioactive molecules production.

	Antagonisitc activity								Bioactive molecules		
Isolate	*F. graminearum*		*Z. tritici*		*G. graminis* var. tritici		*O. yallundae*		Protease	Cellulase	Siderophore
CR6PS3	5.5	^**^	7.2	^***^	11.0	^****^	15.0	^***^	+	+	+
AE10PS1	5.8	^**^	0.0	ns	7.2	^**^	17.7	^****^	+	+	+
BE11PS1	5.8	^***^	0.0	ns	4.7	^***^	13.7	^****^	+	+	+
BF6PS1	4.7	^*^	5.3	^*^	14.2	^***^	14.0	^***^	+	–	+
CR3PS2	3.0	^**^	0.0	ns	13.8	^***^	18.0	^****^	+	–	+
BF10PS1	3.5	^*^	4.8	^***^	14.0	^***^	15.7	^****^	+	+	+
BF8PS1	7.2	^****^	4.3	^****^	10.7	^****^	15.3	^****^	+	+	+
BR8PS4	3.3	^*^	4.7	^***^	13.0	^***^	16.3	^****^	+	+	+
BR8PS5	3.8	^*^	3.8	^****^	10.0	^****^	11.8	^***^	+	+	+
CF11PS1	2.3	ns	0.0	ns	6.0	^**^	6.5	ns	+	+	+
CF10PS4	1.7	ns	3.5	^***^	12.0	^****^	6.5	ns	+	+	+
AE11PS2	5.7	^****^	5.2	^***^	5.2	^**^	13.3	^****^	+	+	+
AE11PS3	5.8	^***^	5.8	^****^	6.8	^**^	16.3	^****^	+	+	+
AE11PS4	6.0	^***^	6.3	^****^	3.0	^***^	15.8	^****^	+	+	+
AE12PS1	7.0	^***^	4.0	^***^	3.8	^**^	13.4	^***^	+	+	+
AE12PS4	4.8	^***^	4.5	^****^	2.8	^**^	15.0	^****^	+	+	+
AE12PS5	3.8	^**^	3.7	^****^	3.3	^*^	14.8	^****^	+	+	+
BS3PS2	3.8	^**^	3.5	^***^	12.7	^****^	16.0	^***^	+	+	+
BS3PS3	3.2	^**^	4.0	^**^	12.7	^****^	14.3	^****^	+	+	+
CE10PS1	3.3	^*^	0.0	ns	3.7	^**^	14.0	^***^	+	+	+
CR6PS4	6.0	^***^	5.8	^**^	9.5	^****^	14.0	^***^	+	+	+
CR6PS5	6.0	^***^	5.2	^**^	10.5	^****^	13.0	^***^	+	+	+
DR10PS5	5.0	^*^	0.0	ns	6.5	^**^	14.7	^****^	+	+	+
AR12PS3	2.8	ns	0.0	ns	17.8	^***^	23.7	^****^	+	+	+
BE10PS1	3.2	^***^	0.0	ns	3.2	^****^	13.3	^****^	+	+	+
BE10PS3	3.1	^***^	0.0	ns	3.2	^****^	13.1	^****^	+	+	+
CF10PS3	1.7	ns	2.7	^***^	5.7	^**^	7.3	ns	+	+	+
CR7PS1	5.8	^**^	0.0	ns	7.0	^**^	17.3	^****^	+	+	+
DR1PS3	3.5	^*^	0.0	ns	9.4	^**^	7.2	ns	+	+	+
DR4PS3	5.3	^**^	5.8	^***^	9.0	^****^	16.6	^****^	+	+	+
DS3PS4	2.7	^**^	0.0	ns	8.5	^***^	19.3	^****^	+	+	+
DS3PS5	4.3	^*^	0.0	ns	8.0	^****^	18.0	^****^	+	+	+
AR11PS3	2.5	^*^	0.0	ns	15.8	^****^	18.7	^****^	+	+	+

**Notes.**

The numbers represent the mean inhibition zone radius in millimeters (*n* = 6), and stars denote the significance of the measured inhibition. Significance was evaluated by comparing measures with the negative control in the Student *t*-test.

ns not significant

* *p*-value <0,5.

** *p*-value <0,01.

*** *p*-value <0,001.

*** *p*-value <0,0001.

All isolates were able to produce siderophores, protease, and cellulase, except for isolates BF6PS3 and CR3PS2 (*Pseudomonas germanica* and *Pseudomonas granadensis*, respectively), which did not exhibit cellulase production. Swimming, swarming, and biofilm formation were specifically assessed for strain CF10PS3, which tested positive for all three traits. These assays were not performed on the other isolates.

MLSA identification based on 10 taxonomically relevant genes compared to the NCBI representative genome database allowed retrieving 11 *Pseudomonas* species in this wheat-associated community (Table 4). *Pseudomonas fluorescens*, *Pseudomonas lurida*, *Pseudomonas poae*, *Pseudomonas salmasensis*, *Pseudomonas orientalis*, and *Pseudomonas simiae* are known to colonize wheat, some strains of these species being known as potential biocontrol agents. On the other hand, *Pseudomonas tensigenes*, *Pseudomonas granadensis*, *Pseudomonas germanica*, *Pseudomonas lactis*, and *Pseudomonas sivasensis* had never been associated with wheat or cereal, to the best of our knowledge. This is particularly interesting for *P. sivasensis* and closely related species since they represent more than 25% of the 33 isolates (Table 4). These isolates originate from all plant compartments and were retrieved from the first sampling which occurred at the end of March until the last sampling which took place at the end of June. They were obtained from the four fields no matter the tillage use and agricultural practices (whether organic or conventional) nor the plant host (spelt or winter wheat).

**Table 4:** Identity percentages between 10 isolate genes used in the MLSA approach and their best-match in the NCBI representative genome database, with the information about isolate origin.

Isolate	Identification based on MLSA against refseq representative genomes database	Type-strain match	% ID with type-strain	Plant organ	Field location	Host	Tillage	Agric. practices	Sampling date
CR6PS3	*P. canadensis*	GZH03	99,79	Root	Walhain	S	No	O	29/04/19
AE10PS1	*P. fluorescens*	ATCC13525	96,95	Head	J-s-S	WW	Yes	O	19/06/19
BE11PS1	*P. fluorescens*	ATCC13525	97,57	Head	Jodoigne	WW	Yes	C	18/06/19
BF6PS1	*P. germanica*	FIT28	99,04	Leaf	Jodoigne	WW	Yes	C	24/04/19
CR3PS2	*P. granadensis*	LMG27940	99,42	Root	Walhain	S	No	O	27/03/19
BF10PS1	*P. lurida*	LMG21995	98,31	Leaf	Jodoigne	WW	Yes	C	18/06/19
BF8PS1	*P. lurida*	LMG21995	99,04	Leaf	Jodoigne	WW	Yes	C	21/05/19
BR8PS4	*P. lurida*	LMG21995	99,34	Root	Jodoigne	WW	Yes	C	21/05/19
BR8PS5	*P. lurida*	LMG21995	98,80	Root	Jodoigne	WW	Yes	C	21/05/19
CF11PS1	*P. orientalis*	8B	97,75	Leaf	Walhain	S	No	O	20/06/19
CF10PS4	*P. poae*	RE^*^1-1-14	98,99	Leaf	Walhain	S	No	O	20/06/19
AE11PS2	*P. salmasensis*	SWRI126	97,77	Head	J-s-S	WW	Yes	O	19/06/19
AE11PS3	*P. salmasensis*	SWRI126	97,37	Head	J-s-S	WW	Yes	O	19/06/19
AE11PS4	*P. salmasensis*	SWRI126	99,5	Head	J-s-S	WW	Yes	O	19/06/19
AE12PS1	*P. salmasensis*	LMG21611	98,79	Head	J-s-S	WW	Yes	O	19/06/19
AE12PS4	*P. salmasensis*	SWRRI126	99,32	Head	J-s-S	WW	Yes	O	19/06/19
AE12PS5	*P. salmasensis*	SWRRI126	99,56	Head	J-s-S	WW	Yes	O	19/06/19
BS3PS2	*P. simiae*	PCL1751	97,21	Soil	Jodoigne	WW	Yes	C	25/03/19
BS3PS3	*P. simiae*	PCL1751	99,32	Soil	Jodoigne	WW	Yes	C	25/03/19
CE10PS1	*P. simiae*	PCL1751	99,2	Head	Walhain	S	No	O	20/06/19
CR6PS4	*P. simiae*	PCL1751	99,71	Root	Walhain	S	No	O	29/04/19
CR6PS5	*P. simiae*	PCL1751	99,57	Root	Walhain	S	No	O	29/04/19
DR10PS5	*P. simiae*	PCL1751	99,53	Root	MSG	WW	No	C	14/06/19
AR12PS3	P. sivasensis^*^ (tmp)	BsEB-1	93,82	Root	J-s-S	WW	Yes	O	19/06/19
BE10PS1	*P. sivasensis*	BsEB-1	98,33	Head	Jodoigne	WW	Yes	C	18/06/19
BE10PS3	*P. sivasensis*	BsEB-1	99,29	Head	Jodoigne	WW	Yes	C	18/06/19
CF10PS3	*P. sivasensis*	BsEB-1	98,9	Leaf	Walhain	S	No	O	20/06/19
CR7PS1	P. sivasensis^**^ (tmp)	BsEB-1	97,39	Root	Walhain	S	No	O	23/05/19
DR1PS3	P. sivasensis^**^ (tmp)	BsEB-1	99,22	Root	MSG	WW	No	C	01/04/19
DR4PS3	P. sivasensis^***^ (tmp)	BsEB-1	99,79	Root	MSG	WW	No	C	25/04/19
DS3PS4	P. sivasensis^**^ (tmp)	BsEB-1	98,67	Soil	MSG	WW	No	C	01/04/19
DS3PS5	P. sivasensis^**^ (tmp)	BsEB-1	99,11	Soil	MSG	WW	No	C	01/04/19
AR11PS3	*P. tensigenes*	ZA 5.3	96,58	Root	J-s-S	WW	Yes	O	19/06/19

**Notes.**

MSG Mont-Saint-Guibert J-s-S Jemeppes-sur-Sambre WW winter wheat S spelt O organic C conventionnel (tmp) Strain initially identified as *P. sivasensis* via MLSA, subject to reidentification as *P. cyclaminis*

*, **, *** (tmp) Strain initially identified as *P. sivasensis via* MLSA, subject to reidentification as *P. cyclaminis* (*), *P. arvensis* sp. nov. (**) or *P. marginalis* (***) - see below - .

### Genome sequencing

Considering the unexpected prevalence of *P. sivasensis* isolates in this community, whole genomes of the nine strains were sequenced using a MinION device and the V14 sequencing chemistry. The genome sequences are deposited on NCBI under BioProject reference number PRJNA1141912 and the strains are deposited in the LMG bacterial collection in Gent, Belgium, with accession numbers provided in Table 5. Strain DR1PS3 was also deposited in DSM collection, Germany, under accession number DSM 118704.

**Table 5:** Genomic characteristics of 11 *P. sivasensis* strains.

**Strain**	**BsEB-1**	**W6**	**CF10PS3**	**BE10PS3**	**BE10PS1**	**CR7PS1**	**DR1PS3**	**DS3PS5**	**DS3PS4**	**AR12PS3**	**DR4PS3**
**LMG accession**	/	/	S-33705	33707	33706	33710	33711	33712	S-33704	33708	33709
**NCBI accession**	SAMN23530418	SAMN15393973	SAMN42904002	SAMN42493717	SAMN42493716	SAMN42493718	SAMN42493719	SAMN42493721	SAMN43319231	SAMN42493722	SAMN42493720
**Size (Mbp)**	6.101	6.109	6.351	6.279	6.279	6.305	6.408	6.246	6.246	6.448	6.576
**Percent G+C**	59.8	59.8	59.6	59.7	59.7	60.2	60.1	60.2	60.2	60.4	60.6
**Protein number**	5463	5506	5687	5609	5611	5659	5729	5568	5573	5744	5880

All 11 genome assemblies, including reference strains BsEB-1 and W6, have one contig with a similar length of 6.30 ± 0.14 Mbp, a GC content of 60.02 ± 0.32%, and 5,639 ± 118 proteins (Table 5). No plasmid was retrieved in the genomic contigs.

**Figure 1:**
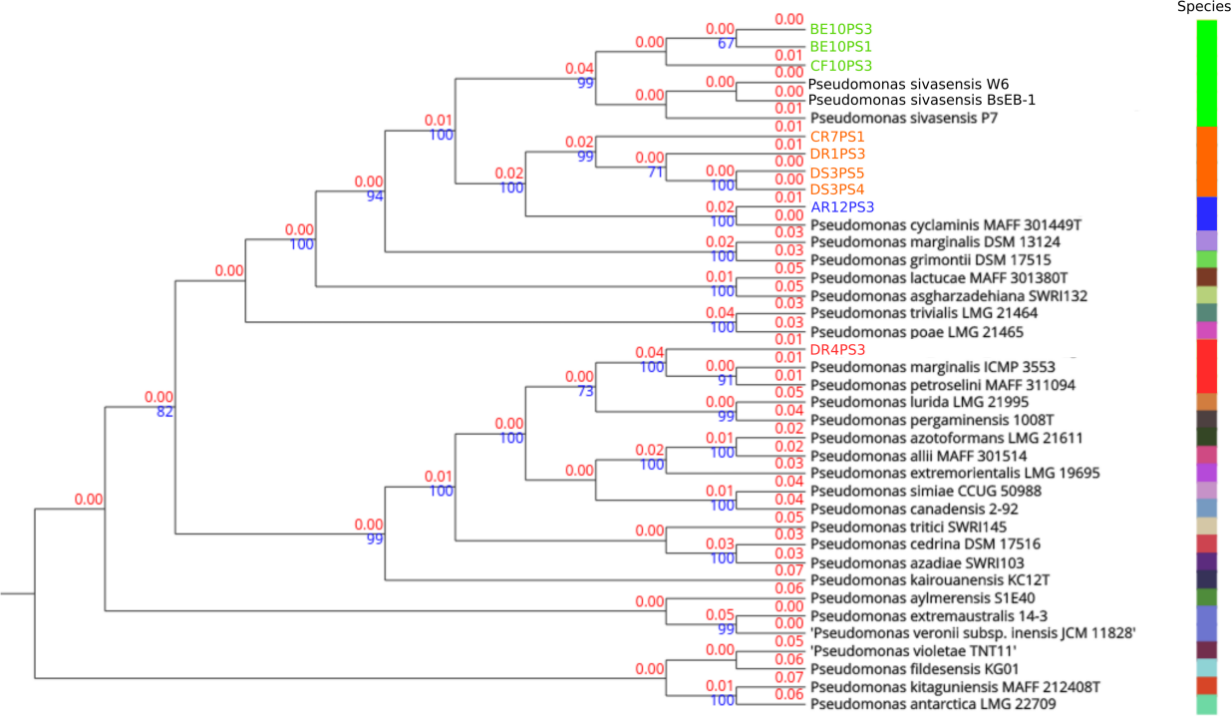
Phylogenetic tree based on complete genome alignments. Tree was build using FastME 2.1.6.1 in TYGS. All query genomes were aligned with the 50 most closely related genomes among the 20,886 genomes in the database. Closest alignments were selected to build the tree. Red numbers are the branch length value, scaled in terms of GBDP distance. The blue numbers above branches are GBDP pseudo-bootstrap support values > 60% from 100 replications, with an average branch support of 79.9%. The tree was rooted at the midpoint. Species delineation is represented by the color bar on the right.

In-depth genome alignments performed *via* TYGS delineated phylogenetic relationships across 27 distinct species clusters, as illustrated in Fig. 1. Four clusters were assigned to the query strains, indicating notable genetic delineations. Notably, in TYGS tool strain P7 is the reference genome for *P. sivasensis*, which closely grouped with two additional queried reference genomes, BsEB-1 and W6, alongside with strains BE10PS1, BE10PS3, and CF10PS3, as evidenced by short branch lengths indicating minor genetic distances (red numbers). Conversely, strains CR7PS1, DR1PS3, DS3PS4, and DS3PS5 formed a distinct cluster, characterized by longer branch lengths from known species, suggesting a potential new species. This hypothesis was further supported by robust bootstrap values above 70% (blue numbers), confirming the reliability of these phylogenetic relationships. Additionally, AR12PS3 and DR4PS3 showed close affiliations with *P. cyclaminis* and *P. marginalis*, respectively.

**Figure 2:**
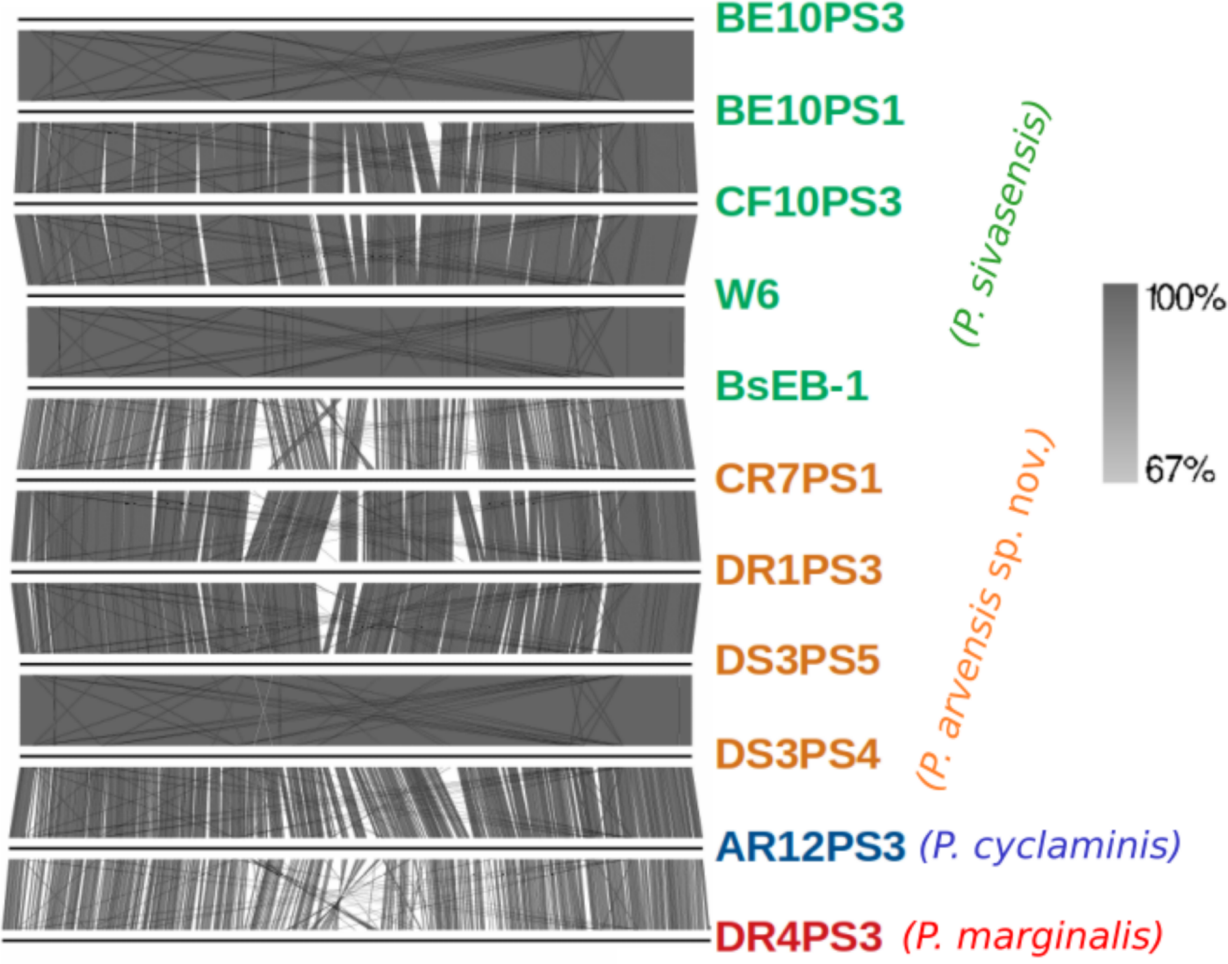
Genome organization of sequenced strains. Plain horizontal lines represent the complete aligned genomes. Orthologous clusters between genomes are connected by shades of gray indicating the percentage of similarity, as depicted in the right part.

Detailed comparison of complete genome organization across strains, as visualized in Fig. 2, aligned consistently with phylogenetic relationships established by TYGS. Each genome is marked distinctly, with *P. sivasensis* genomes shown in green, illustrating homogeneous genomic structures indicative of close evolutionary relationships. In contrast, genomes attributed to a *P. arvensis* sp. nov. demonstrated a unique organizational pattern, emphasizing potential genomic divergence. Notably, the comparison between the BsEB-1 and CR7PS1 genomes highlighted significant organizational variations. Additionally, the genome of *P. cyclaminis* AR12PS3 was more similar to that of the new species than that of *P. sivasensis* in its organization, suggesting nuanced evolutionary relationships. In contrast, *P. marginalis* DR4PS3 exhibits distinct genomic features, underscoring its phylogenetic distance.

### Genome mining

In the results presented in Table 6, the genomic comparisons between various strains of *P. sivasensis* and related species were quantified using orthoANIu and dDDH metrics. The orthoANIu values, typically ranging above 99% among closely related *P. sivasensis* strains (BsEB-1, W6, CF10PS3, BE10PS1 and BE10PS3), underscore a strong genetic relatedness. Conversely, dDDH values, crucial for delineating species boundaries, highlighted more pronounced differences, especially when comparing *P. sivasensis* with distinct species such as *P. cyclaminis* and *P. marginalis*. The lower dDDH values between these species, often below the 70% species delineation threshold, support their classification as separate species. The genomic analysis reinforces that strain AR12PS3 is close to *P. cyclaminis* strain MAFF 301449T and the strain DR4PS3 to *P. marginalis* ICMP 3553, given their ANI and dDDH values (AR12PS3: 98.96%, 91.4%; DR4PS3: 98.87%, 90.0%). The proposed new *Pseudomonas* species comprising strains CR7PS1, DR1PS3, DS3PS4, and DS3PS5 showed high intra-group ANI values exceeding 98.82%, signifying close genetic relationship. Although their ANI values with *P. cyclaminis* stood at approximately 95.80%, just under the threshold (Rosselló-Móra & Amann, 2015), their dDDH values with a species representative genome or with the isolate AR12PS3 ranged from 65 to 66%, below the 70% species delimitation threshold (Meier-Kolthoff et al., 2013).

**Table 6:** OrthoANIu and dDDH values calculated between genomes.

			orthoANIu												
			*P. sivasensis*					*P. arvensis* sp. nov.				*P. cyclaminis*	*P. marginalis*	*P. cyclaminis*	*P. marginalis*
			BsEB-1	W6	CF10PS3	BE10PS3	BE10PS1	CR7PS1	DR1PS3	DS3PS5	DS3PS4	AR12PS3	DR4PS3	MAFF 301449T	ICMP 3553
															
dDDH	*P. sivasensis*	BsEB-1	/	99.98	98.93	99.05	99.05	91.63	91.65	91.69	91.69	91.69	88.14	91.36	87.79
		W6	100	/	99.06	99.04	99.07	91.42	91.37	91.29	91.28	91.52	87.92	91.6	87.85
		CF10PS3	91.8	91.7	/	99.06	99.05	91.43	91.33	91.28	91.28	91.52	87.78	91.52	89.55
		BE10PS3	91.9	91.8	92	/	99.99	91.25	91.38	91.56	91.56	91.46	88.04	91.48	89.56
		BE10PS1	91.9	91.8	92	100	/	91.27	91.44	91.52	91.52	91.46	87.99	91.53	89.56
	*P. arvensis* sp. nov.	CR7PS1	44.4	44.4	44.3	44.6	44.6	/	98.82	98.88	98.89	95.78	88.1	95.76	89.54
		DR1PS3	44.3	44.3	44.2	44.6	44.6	91	/	99.05	99.05	95.78	88.09	95.78	89.55
		DS3PS5	44.3	44.3	44.3	44.5	44.5	91.6	92	/	99.99	95.86	88.14	95.81	89.52
		DS3PS4	44.3	44.3	44.3	44.5	44.5	91.6	92	100	/	95.86	88.15	95.8	89.52
	*P. cyclaminis*	AR12PS3	44.4	44.4	44.5	44.5	44.5	66.1	65.6	65.9	65.9	/	88.17	98.96	89.58
	*P. marginalis*	DR4PS3	34.5	34.5	34.7	34.8	34.8	35.1	35	35	35	35.4	/	88.26	98.87
	*P. cyclaminis*	MAFF 301449T	44.7	44.7	44.7	44.8	44.8	66.1	65.2	65.5	65.5	91.4	35.5	/	88.12
	*P. marginalis*	ICMP 3553	34.5	34.5	34.6	34.8	34.8	35.1	35	35	35	35.3	90	/	/

**Notes.**

OrthoANIu values are in the upper right part and dDDH values in the lower left part.

**Figure 3:**
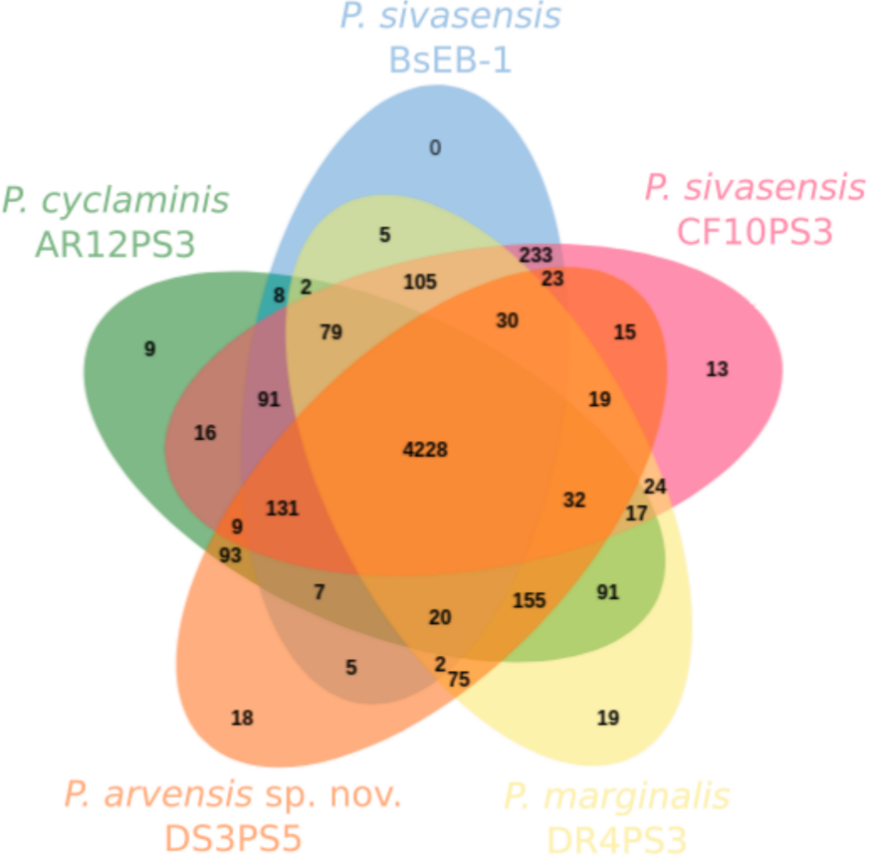
Core and pan-genome analysis of one strain of each species compared with *P. sivasensis* reference strain (BsEB-1). Venn diagram of orthologous clusters generated by OrthoVenn3.

Figure 3 illustrates the core and pan-genome of *P. sivasensis* and related strains, using a Venn diagram to depict orthologous gene clusters. For the sake of clarity, one isolate of each species and the reference genome BsEB-1 were used. This analysis provides insights into the genetic conservation and variability within these strains, highlighting the core genes essential for basic cellular functions and shared across all examined strains. Additionally, it also delineates genes unique to specific strains, reflecting their evolutionary adaptations and potential specialized functions. The pan-genome size indicates the total genetic diversity found across all strains, essential for understanding the species’ adaptability and ecological fitness.

In this analysis, 4,950 ± 76 clusters were detected within each genome. Of these, 4,228 were shared across all five isolates. Additionally, 233 clusters were exclusively shared by the two representatives of *P. sivasensis*, BsEB-1 and CF10PS3. The later strain harbored 13 unique clusters, 12 of which being of unknown function, while the remaining one contained the *GspE* gene, involved in the type II secretion system. Strains DR4PS3, DS3PS5, and AR12PS3 possessed 19, 18, and nine unique clusters, respectively. These clusters were predominantly of unknown function, except in the case of AR12PS3, where processes related to flavonoid and macrolide biosynthesis were identified, along with a fimbrial protein associated with cell adhesion.

**Figure 4:**
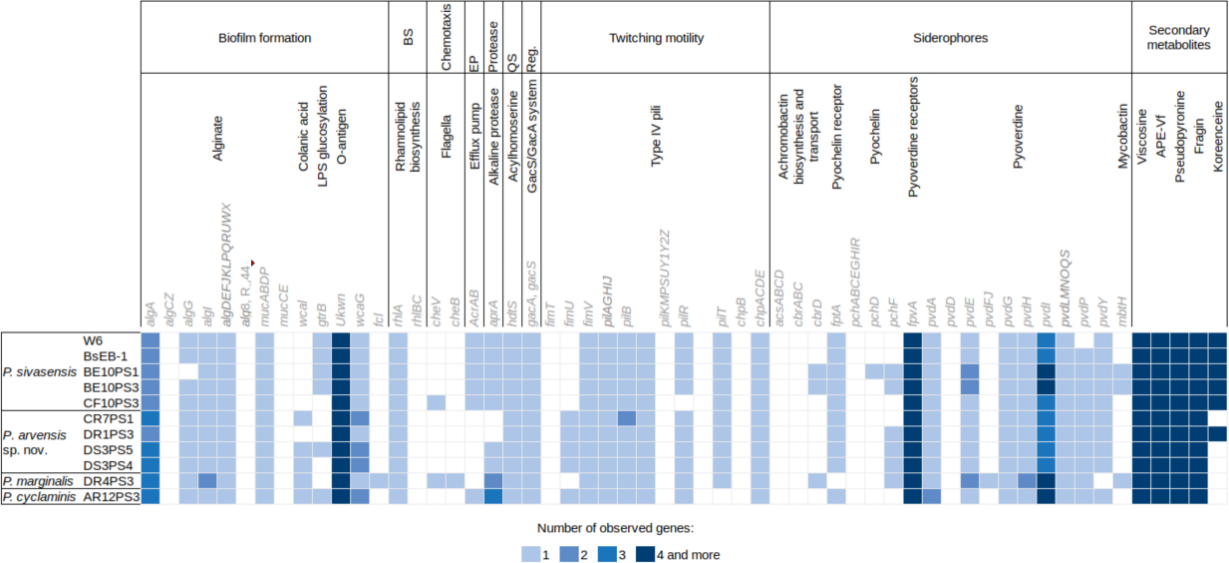
Distribution of genes potentially involved in biocontrol in the nine sequenced genomes and two *P. sivasensis* reference genomes.

Figure 4 illustrates the genetic profiles of each strain concerning their biocontrol potential. Here, we present a subset of biocontrol-related genes from the VFdb and ANTISMASH databases, focusing on genes for which some strains showed a match. There were no discernible trends in the presence or absence of genes among different species. However, one notable exception is the presence of the efflux pump encoded by *AcrAB* genes, which was exclusively found in *P. sivasensis* strains and *P. cyclaminis* AR12PS3. Notably, *P. sivasensis*, along with *P. arvensis* sp. nov. DR1PS3, possessed the complete operon for koreenceine production. All genomes analyzed in this study contained operons for viscosin, APE-Vf, fragin and pseudopyronine production. Conversely, the *pvdD* gene, a key component of the pyoverdine biosynthetic gene cluster, was not identified in any genomes, although all strains possessed the *fpvA* gene which encodes the pyoverdine receptor. Similarly, none of the strains possessed a complete operon for pyochelin production, but all except DR4PS3 had the *fptA* gene encoding the pyochelin receptor. Notably, all strains exhibited potential quorum sensing capability through Acyl-homoserine lactone and possessed the *GacA/GacS* regulatory system.

### Description of *Pseudomonas arvensis* sp. nov.

*Pseudomonas arvensis* (ar.ven’sis. L. adj. arvensis, “of fields”) is a bacterium isolated from agricultural soils. Cells are aerobic, Gram-negative, rod-shaped, and exhibit polar flagellation. The bacterium is oxidase- and catalase-positive. Colonies grown on King’s B agar are round, flat, opaque, beige, with regular margins, and reach approximately two mm in diameter after 2 days of incubation at 28 °C. When cultured on King’s B agar or CFC agar (cephaloridine, fusidic acid, and cetrimide), colonies fluoresce under UV light. The bacterium grows in the presence of 0–6% NaCl, at pH 5–8, and at temperatures ranging from 4 to 30 °C.

Biolog plate assays indicate the ability to oxidize a variety of carbon sources, including 4-hydroxybenzoic acid, D-galactonic acid *γ*-lactone, D-galacturonic acid, D-glucosaminic acid, D-mannitol, D-xylose, D,L-*α*-glycerol phosphate, L-arginine, L-asparagine, L-serine, pyruvic acid methyl ester, Tween 40, Tween 80, *β*-hydroxy-glycyl-L-glutamic acid, and *γ*-aminobutyric acid. API 20NE galleries reveal the ability to reduce nitrate to nitrogen, and to utilize D-glucose, L-arabinose, D-mannose, D-mannitol, N-acetylglucosamine, potassium gluconate, capric acid, malic acid, and trisodium citrate as substrates.

Tests for bioactive compound production, along with genome mining, reveal the capacity to produce protease, cellulase, and siderophores. The genome encodes complete operons for the biosynthesis of fragin, viscosin, APE-Vf, pseudopyronine, and acyl-homoserine lactones, suggesting potential for quorum sensing and the production of diverse bioactive metabolites. The strain demonstrates the ability to translocate from seed coatings to plant leaves *via* hydathodes, highlighting its ecological and agricultural relevance.

The type strain, DR1PS3 (=UPB1363 =LMG 33711 =DSM 118704), was isolated from the rhizosphere of winter wheat in a conventionally managed agricultural field in Mont-Saint-Guibert, Belgium (50°39′08.2″N, 4°38′20.4″E; 130 m above sea level), sampled on 1 April 2019. The DNA G+C content is 60.1%, and the genome size is 6.408 Mbp, encoding 5,729 predicted proteins. The whole-genome sequencing project for strain DR1PS3 has been deposited in GenBank under the accession number CP176438. Phylogenetically, *Pseudomonas arvensis* is closely related to *Pseudomonas cyclaminis* and *Pseudomonas sivasensis*. Digital DNA–DNA hybridization (dDDH) values with *P. cyclaminis* MAFF 301449 and *P. sivasensis* BsEB-1 are 65.2% and 44.3%, respectively, while OrthoANIu values are 95.78% and 91.65%, supporting its designation as a novel species.

### Plant colonization

**Figure 5:**
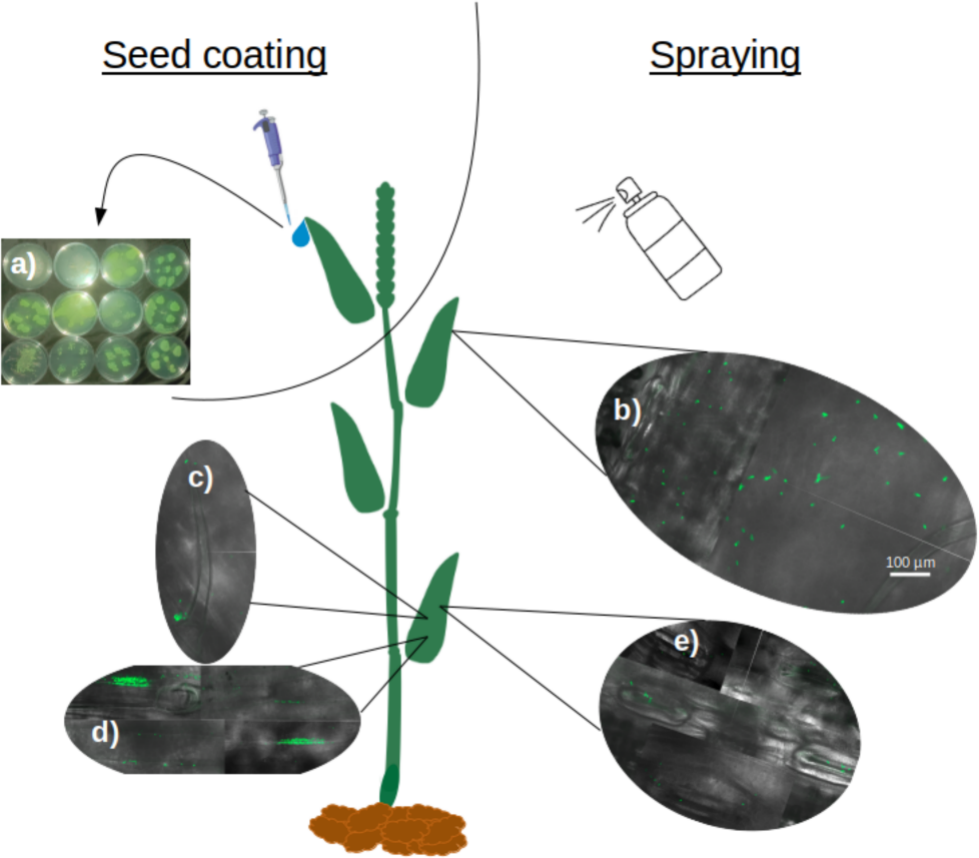
Translocation and colonization abilities of *Pseudomonas* strains. (A) Exit of all tested strains through hydathodes after translocation from seed, visualized by spreading hydathode droplets on Petri dishes with CFC medium (bacteria applied exclusively *via* seed coating). (B–E) Colonization pattern of CF10PS3 on wheat leaves visualized by confocal microscopy of GFP-expressing cells: (B) initial homogeneous distribution of single cells after foliar spray; (C) aggregation on trichomes and their bases; (D) colonization of veins; (E) preferential localization around stomata. All confocal images were taken at 630X magnification and share the same scale bar shown in (B).

Soaking wheat seeds in bacterial suspensions of all nine strains of *P. sivasensis* and closely related species, followed by immediate sowing, enabled the bacteria to colonize the seedlings and translocate to the aerial parts. This was demonstrated by recovering bacteria from morning dew droplets collected in the days following seedling emergence, as evidenced by their growth and fluorescence on CFC agar plates (Fig. 5A). Among *P. sivasensis* strains, CF10PS3 was isolated from leaf and was able to antagonize *Z. tritici* during *in vitro* tests. Its GFP-expressing mutant was used in subsequent tests to evaluate its plant colonization abilities. Following seed inoculation, CF10PS3 was transported through xylem and exited leaves through hydathodes. From there, it was able to colonize the leaf surface. As shown with confocal imagery after leaf spraying, CF10PS3 can thrive on leaf surface. Immediately following application, single bacteria were found homogeneously distributed on the surface (Fig. 5B). Two days later, it was found in large aggregates that colonized trichomes and their base (Fig. 5C), veins (Fig. 5D) and more importantly, they occupied the niche around stomata (Fig. 5E).

### Plant protection

**Figure 6:**
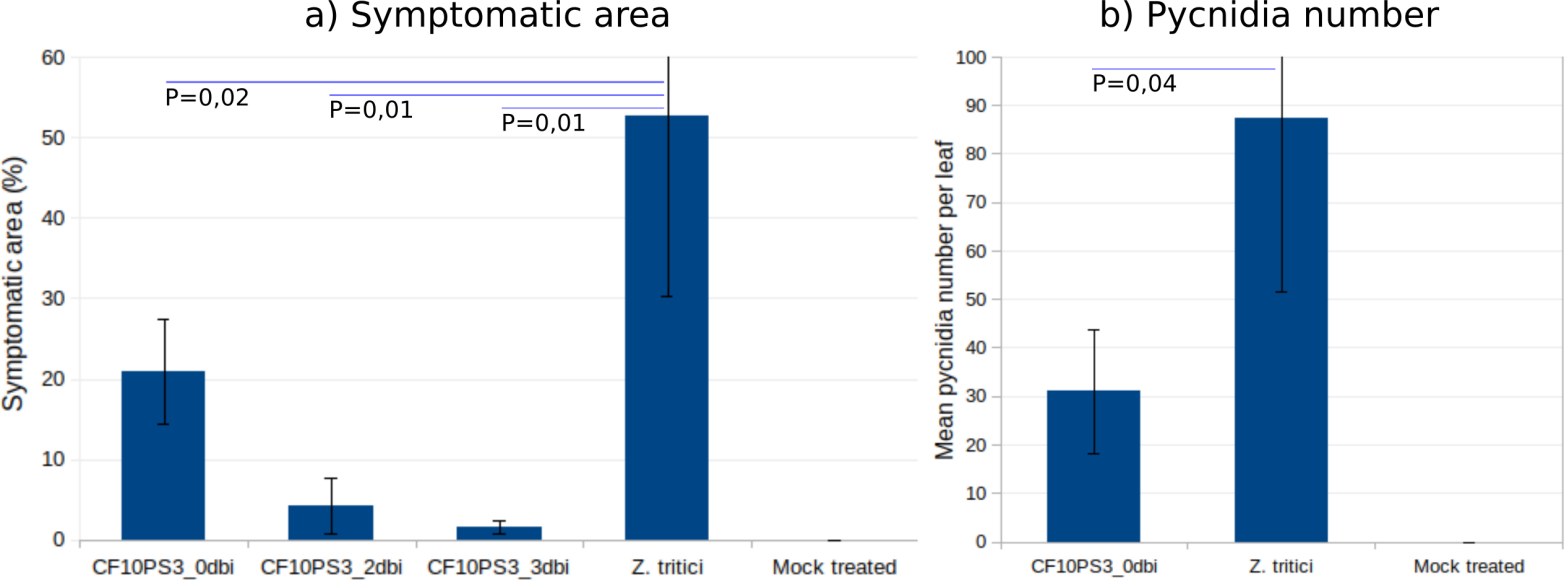
*Zymoseptoria tritici* control by CF10PS3 treatment. (A) symptomatic area on leaves in boxes where bacteria was applied 3 days before pathogen inoculation (3 dbi), 2 dbi or the same day than *Z. tritici* conidia (0 dbi). (B) Mean pycnidia number per leaf produced by the lesions with or without CF10PS3 application. Student test *P* values are indicated (*n* = 8). Controls were inoculated with *Z. tritici* conidia only, whereas mock treatment were sprayed with the buffer only.

CF10PS3 also demonstrated the capability to reduce the symptomatic area of *Z. tritici*. In boxes where *Z. tritici* was applied alone to evaluate the infection level without protection, leaf area covered by lesion was about 50% (Fig. 6A) and mean pycnidia number produced by these lesions on each leaf was 87.3 (Fig. 6B). When CF10PS3 was applied just before the pathogen, symptomatic area decreased to about 20% and mean pycnidia number dropped to 31. This effect was even more noticeable when applied two and three days before infection (dbi), with symptomatic area of 5 and 2%, respectively. Considering these low infection levels, no pycnidia were produced on the few symptomatic tissues.

## Discussion

The *Pseudomonas* genus, known for its significance in association with a variety of plants (Preston, 2004; Poncheewin et al., 2022), and particularly cereals (Mehmood et al., 2023), has once again demonstrated its potential in biocontrol. In this study, from 444 isolates obtained in several cereal fields in Belgium, 33 showed biological activity against at least three of the four wheat pathogens tested (*F. graminearum*, *G. graminis* var. tritici, *O. yallundae*, and *Z. tritici*), underscoring the biocontrol potential of this genus.

Recent studies, including the present work, have expanded the recognized diversity and functional repertoire of *Pseudomonas* associated with wheat. Poli, Keel & Garrido-Sanz (2024) similarly described four novel *Pseudomonas* species from the wheat rhizosphere with demonstrated antagonism toward key fungal pathogens and direct growth-promoting activities, echoing our findings with *P. arvensis* sp. nov. and *P. sivasensis*. Several studies highlight the molecular and ecological mechanisms underlying these benefits: Guo et al. (2024) identified *P. khavaziana* SR9 as a highly effective biocontrol agent against wheat crown rot, mediated by phenazine biosynthesis, while Guan et al. (2024) demonstrated that the production of cyclic lipopeptides such as viscosin not only enhances wheat root colonization but also modulates the root microbiome in a plant genotype-dependent manner. Field trials with *Pseudomonas* consortia (Dobrzyński et al., 2025) and stress-tolerant *P. guariconensis* strains (Kankariya et al., 2024) confirm that these bacteria can boost yield and plant resilience under real-world conditions, often without major disruption to the native microbial community. Collectively, these studies, including our own, underscore the wheat microbiome as a rich source of *Pseudomonas* diversity and biocontrol agents.

Initially, in the present work, Sanger sequencing of 10 taxonomically relevant genes (16S rRNA, *gapA, glnS, gltA, gyrB, ileS, nuoD, recA, rpoB,* and *rpoD*) through a Multi-Locus Sequence Analysis (MLSA) approach allowed for the identification of 11 species. Among these, *P. fluorescens*, *P. lurida*, *P. poae*, *P. salmasensis*, *P. orientalis*, and *P. simiae* are known to colonize wheat, some strains of these species being known as potential biocontrol agents (Kron et al., 2020; Gómez-Lama Cabanás et al., 2022; Ibrahim et al., 2023). In addition, isolates have also been identified as *P. tensinigenes*, known to colonize wheat rhizosphere and produce tensin (Girard et al., 2021), *P. granadensis* already isolated from soil (Pascual et al., 2015), *P. germanica* obtained from *Iris germanica* rhizomes (Atanasov et al., 2022), known soil inhabitant *P. lactis* (Havryliuk et al., 2020), and *P. sivasensis*. The MLSA reported nine isolates, more than one quarter, as belonging to *P. sivasensis* with high percentages of identity, greater than 97%, except for isolate AR12PS3 for which the best match was still *P. sivasensis*, but with a lower 93% identity. *P. sivasensis* was known to promote the growth of canola (Świa̧tczak, Kalwasińska & Brzezinska, 2023) and was spotted in different environments, ranging from the *Bletilla striata* (hyacinth orchid) root endosphere (Wu et al., 2022) to aquatic or industrial environments (Duman et al., 2020), as well as in a high plateau wetland ecosystem (Xiong et al., 2023) but was never associated with cereals, to the best of our knowledge. Its high prevalence in the four fields and all plant compartments observed with the samplings encouraged us to investigate further.

Whole genome sequencing of these nine strains confirmed only three of the isolates as true *P. sivasensis*, despite initial MLSA findings. This highlights the superior resolution of WGS over MLSA in species identification (Mulet et al., 2023). The remaining isolates were reclassified; one as *P. cyclaminis* (AR12PS3), a species phylogenetically close to *P. marginalis* and *P. grimontii* (Sawada et al., 2021), and another as *P. marginalis* (DR4PS3). The other four could constitute a new species, with the proposed name *Pseudomonas arvensis* sp. nov., with DR1PS3 as the type-strain and strains CR7PS1, DS3PS4, and DS3PS5 as other representatives. These isolates did not exhibit any plant pathogenic behaviors and were able to antagonize the wheat pathogens tested in this study, to varying degrees. This new species, phylogenetically closer to *P. cyclaminis* and close to *P. sivasensis*, *P. marginalis*, and *P. grimontii*, presents an interesting case with Average Nucleotide Identity values calculated with orthoANIu method, considered as the most accurate method (Yoon et al., 2017)—around 95.80% and digital DNA-DNA Hybridization values between 65 to 66%, just below the ANI and dDDH thresholds commonly used for species delineation (96% and 70%, respectively) (Madhaiyan et al., 2017). The close evolutionary relationships among these species are reflected in their genomic structure and gene composition, with 4,228 of 4,950 gene clusters being common across the genomes studied. Significantly, the conserved regions comprise operons responsible for the production of viscosin, APE-Vf, pseudopyronine, and acyl-homoserine lactone production. Viscosin is a lipopeptidic biosurfactant involved in motility and biofilms, and harboring PGP traits and antifungal properties (Alsohim et al., 2014). APE-Vf is an arylpolyene with antifungal and biocontrol activities in *P. fluorescens NBC275* (Dutta, Yu & Lee, 2020). Pseudopyronine is a pyrone with significant activity against agriculturally important fungi (Nishanth Kumar et al., 2016). The diazeniumdiolate secondary metabolite fragin is a metallophore with reported antifungal activity (Jenul et al., 2018). Acyl-homoserine lactone (AHL) is a quorum-sensing (QS) signaling molecule with a potent effect on pathogenic microbes on leaves (Chaudhry et al., 2021). All *P. sivasensis* strains and *P. arvensis* sp. nov. strain DR1PS3 shared the complete operon for koreenceine production, a polyketide with antifungal effects (Riera et al., 2023). Although genome mining did not reveal complete operons for the canonical siderophores pyoverdine and pyochelin, all strains produced siderophores *in vitro*, likely *via* alternative pathways. The presence of biosynthetic gene clusters for other metallophores, such as fragin, and of genes encoding siderophore receptors (fpvA, fptA) supports the existence of functional siderophore systems. Additionally, it is possible that divergent or uncharacterized siderophore biosynthetic pathways contribute to the observed activity.

Among the strains, *P. sivasensis* CF10PS3 was isolated from leaf and demonstrated notable *in vitro* antagonistic activity against *Z. tritici*. It was further explored for its plant colonization capabilities. Following seed inoculation, CF10PS3 was demonstrated to move *via* the xylem, exited leaves through hydathodes, and demonstrated effective colonization of leaf surfaces. It was shown that the strain will preferentially establish itself around stomata, along the veins, and at the bases of trichomes, considered as preferential sites for microbial colonization as reviewed by Vacher et al. (2016). Translocation and proliferation of *Pseudomonas* in plants have already been highlighted, notably with *P. chlororaphis* (Hernandez-Jerez et al., 2020). These bacteria applied to seeds could indeed migrate from the seed to various plant tissues, including roots and foliage, albeit often at lower concentrations in the foliage. Other studies have also demonstrated that *P. chlororaphis* can internally colonize plant tissues, indicating that seedborne applications can effectively distribute the biocontrol agent within the plant (Mercado-Blanco & Bakker, 2007; Finkel et al., 2017). This internalization suggests that the bacteria not only adhere to external plant surfaces but can also penetrate and establish within, utilizing pathways like root hairs, cracks, and wounds. This movement is crucial for the bacteria’s role in biocontrol, enhancing their effectiveness by inhabiting spaces that are critical for pathogen interaction and plant health support. Following spray application on leaves, confocal microscopy revealed initial homogeneous distribution of bacteria, which then aggregated significantly around trichomes, veins, and stomata within two days. Most epiphytes survive on the leaf surface by forming large aggregates which help them to cope with the surrounding milieu and maintain a hydrated surface by the production of extracellular polymeric substances (Morris & Kinkel, 2002; Lindow & Brandl, 2003; Baldotto & Olivares, 2008; Vorholt, 2012). CF10PS3’s ability to produce hormones and surfactants, as well as its motility and biofilm formation are likely to be key factors in the colonization success (Nadakuduti et al., 2012; Braga, Dourado & Araújo, 2016; Ueda et al., 2018; Leveau, 2019; Oso et al., 2019; Streletskii et al., 2019; Flores-Núñez et al., 2020). This strategic colonization presumably facilitates the observed reduction in symptomatic areas of *Z. tritici* infection and the reduction of pycnidia produced by lesions. Preinfection applications were the most effective. *Z. tritici* conidia germinate on the leaf surface, undergo an epiphytic growth phase (Fones et al., 2017), and then invade the plant through the stomata (Battache et al., 2022). Near the stomata, the fungus can encounters CF10PS3, which can exert its antagonistic effects to reduce the number of hyphae that penetrate the stomata and start an infection.

## Conclusions

This study provides new insights into the diversity and ecological role of *Pseudomonas* species associated with Belgian cereal crops. Through systematic sampling and characterization, we revealed a significant prevalence of *Pseudomonas sivasensis* in wheat and spelt across multiple fields and plant compartments, marking its first documented association with cereal crops. Moreover, whole-genome sequencing enabled the description of *Pseudomonas arvensis* sp. nov., a novel species represented by strain DR1PS3 and closely related strains, enriching our understanding of *Pseudomonas* taxonomy.

Functional assays demonstrated that these *Pseudomonas* isolates, particularly *P. sivasensis* CF10PS3, display potent antagonistic activity against major wheat pathogens, notably *Zymoseptoria tritici*, and are capable of efficient colonization of the phyllosphere, including systemic movement from seed to leaf *via* hydathodes. Genome mining confirmed the presence of multiple biocontrol- and colonization-related gene clusters, further supporting their ecological significance and potential utility as biocontrol agents in sustainable agriculture.

These findings advance our taxonomic and ecological knowledge of cereal-associated *Pseudomonas* and highlight the wheat microbiome as a reservoir of beneficial bacteria with natural biocontrol potential. The discovery of *P. arvensis* sp. nov. underlines the importance of genomic approaches for revealing hidden microbial diversity and function.

Going forward, in-depth investigation into the *in planta* mechanisms, environmental persistence, and interaction networks of these strains will be essential to translate their biocontrol potential into field applications. Additionally, exploring how agricultural practices and environmental variables shape their prevalence and activity could help design microbiome-based strategies for disease management and crop resilience.

## Supplemental Information

10.7717/peerj.20177/supp-1 Supplemental Information 1 Raw data of inhibition tests

10.7717/peerj.20177/supp-2 Supplemental Information 2 Raw data of biocontrol protection against Z. tritici

10.7717/peerj.20177/supp-3 Supplemental Information 3 Type-strain DR1PS3 deposition at BCCM/LMG

10.7717/peerj.20177/supp-4 Supplemental Information 4 Type-strain DR1PS3 deposition at DSMZ

## Additional Information and Declarations

### Data Availability

The following information was supplied regarding data availability: The raw sequence data are available at NCBI BioProject: PRJNA1141912
The genomes are also available at Zenodo: Delitte, M. (2025). 9Genomes_Pseudomonas [Data set]. In PeerJ. Zenodo. https://doi.org/10.5281/zenodo.12743961.

### New Species Registration

The following information was supplied regarding the registration of a newly described species: The reference strain for *Pseudomonas arvensis* is deposited at BCCM/LMG (https://bccm.belspo.be/about-LMG) and DSMZ (https://www.dsmz.de/).
