# Supplementary material for: Biocontrol-relevant diversity of wheat-associated Pseudomonas: prevalence of P. sivasensis and identification of the novel species P. arvensis sp. nov
Source: PeerJ. 2025 Nov 5;13:e20177. doi: 10.7717/peerj.20177 (PMC12595949; doi:10.7717/peerj.20177)
Supplement: Supplemental Information 3 [file peerj-13-20177-s003.pdf]

BCCM · LMG

GREAT AT SMALL THINGS

Gent, August 23, 2024

CERTIFICATE OF DEPOSIT

This is to certify that the following microorganism has been deposited into the public BCCM/LMG Bacteria Collection and will be available for research purposes without restrictions:

**LMG Number:**	**LMG 33711**
**Species name:**	Pseudomonas arvensis
**Depositor:**	Mathieu Delitte, UCL - Earth and Life Institute, ELIM, Belgium
**Depositor no:**	DR1PS3

The strain has been checked for viability and is preserved using one of the standard methods at BCCM/LMG. Authenticity was confirmed by the BCCM/LMG quality control check as communicated with the depositor.

BCCM/LMG is not responsible for eventual discrepancies in characterization of the deposited material and its original description.

Digitally signed by ir Claudine Vereecke, curator
Date: 2024.08.23 09:17:39 +02'00'

BCCM DCG
BCCM GeneCorner
BCCM IHEM
BCCM ITM
BCCM MUCL
BCCM ULC
BCCM CC

BCCM/LMG - Laboratory for Microbiology
Ghent University K.L. Ledeganckstraat 35 9000 Gent Belgium
+32 (0)9 264 51 08 bccm.lmg@ugent.be bccm.belspo.be
@BccmCollections BCCM Collections

GHENT UNIVERSITY Belgian Science Policy Office .be
