# Supplementary material for: Biocontrol-relevant diversity of wheat-associated Pseudomonas: prevalence of P. sivasensis and identification of the novel species P. arvensis sp. nov
Source: PeerJ. 2025 Nov 5;13:e20177. doi: 10.7717/peerj.20177 (PMC12595949; doi:10.7717/peerj.20177)
Supplement: Supplemental Information 4 [file peerj-13-20177-s004.pdf]

Leibniz-Institut
DSMZ-Deutsche Sammlung von
Mikroorganismen und Zellkulturen GmbH

Braunschweig, **13.12.2024**

Confirmation of the availability of a strain for the purpose of valid publication of a new name according to the Prokaryotic Code

The following information is confidential and serves only to allow an international journal to confirm that a strain has been deposited and will be available from the DSMZ in accordance with the Rules of the Prokaryotic Code, ICNP (2022 Revision).

strain DR1PS3

has been deposited in the DSMZ under the number

DSM 118704

This strain has been checked for viability and authenticity and is preserved in the DSMZ.

This strain is available in the publicly accessible section of the DSMZ and restrictions have not been placed on access to information concerning the presence of this strain in the DSMZ. It will be included in published and online catalogues after publication of this number by the authors. Strains collected after October 2014, may be accompanied by the Nagoya Protocol documentation (e.g., PIC, MAT, MTA, or IRCC), which have been reviewed by DSMZ. All strains will be available from the DSMZ in accordance to our standard terms and conditions.

The DSMZ is not responsible for differences between the properties of the strain deposited in the DSMZ and properties given in the literature/databases.

It is the sole responsibility of the depositor to ensure that type strains deposited in the DSMZ conform to the requirements of the appropriate Rules governing prokaryotes nomenclature and the deposition of type strains [Rules 18a, 27, & 30 of the ICNP (2022 Revision)].

Dr. Richard Hahnke   Curator Gram-negative Bacteria

Geschäftsführung/Directors:
Prof. Dr. Jörg Overmann
Bettina Fischer
Aufsichtsratsvorsitzender/Head of
Supervisory Board: MR Dr. David Schnieders

Braunschweigische Landessparkasse
(NORD/LB) Kto.-Nr./Account: 2 039 220
BLZ/Bank Code: 250 500 00
IBAN DE22 2505 0000 0002 0392 20
SWIFT (BIC) NOLADE 2 H

Handelsregister/
Commercial Register:
Amtsgericht Braunschweig
HRB 2570
Steuer-Nr. 13/200/24030

Zertifikat seit 2010 audit berufundfamilie

Abteilung Pflanzenviren:
DAkkS Deutsche Akkreditierungsstelle D-RM-18324-01-00
